# ‘IRDiRC Recognized Resources': a new mechanism to support scientists to conduct efficient, high-quality research for rare diseases

**DOI:** 10.1038/ejhg.2016.137

**Published:** 2016-10-26

**Authors:** Hanns Lochmüller, Yann Le Cam, Anneliene H Jonker, Lilian PL Lau, Gareth Baynam, Petra Kaufmann, Paul Lasko, Hugh JS Dawkins, Christopher P Austin, Kym M Boycott

**Affiliations:** 1 Institute of Genetic Medicine, Newcastle University, Newcastle upon Tyne, UK; 2 European Organisation for Rare Diseases (EURORDIS), Paris, France; 3 IRDiRC Scientific Secretariat, Inserm US 14, Paris, France; 4 Genetic Services of Western Australia, King Edward Memorial Hospital, Perth, Western Australia, Australia; 5 Western Australian Register of Developmental Anomalies, Perth, Western Australia, Australia; 6 National Center for Advancing Translational Sciences, National Institutes of Health, Bethesda, Maryland, USA; 7 Department of Biology, McGill University, Montreal, Quebec, Canada; 8 Department of Health, Office of Population Health Genomics, Public Health Division, Government of Western Australia, Perth, Western Australia, Australia; 9 Department of Genetics, Children's Hospital of Eastern Ontario Research Institute, University of Ottawa, Ottawa, Ontario, Canada

## Abstract

The International Rare Diseases Research Consortium (IRDiRC) has created a quality label, ‘IRDiRC Recognized Resources', formerly known as ‘IRDiRC Recommended'. It is a peer-reviewed quality indicator process established based on the IRDiRC Policies and Guidelines to designate resources (ie, standards, guidelines, tools, and platforms) designed to accelerate the pace of discoveries and translation into clinical applications for the rare disease (RD) research community. In its first year of implementation, 13 resources successfully applied for this designation, each focused on key areas essential to IRDiRC objectives and to the field of RD research more broadly. These included data sharing for discovery, knowledge organisation and ontologies, networking patient registries, and therapeutic development. ‘IRDiRC Recognized Resources' is a mechanism aimed to provide community-approved contributions to RD research higher visibility, and encourage researchers to adopt recognised standards, guidelines, tools, and platforms that facilitate research advances guided by the principles of interoperability and sharing.

The International Rare Diseases Research Consortium (IRDiRC) was launched in 2011 to foster international research collaboration and investment in the field of rare disease (RD), with the objectives to contribute to the development of 200 new therapies and the means to diagnose most RDs by the year 2020. In 2013, IRDiRC issued its Policies and Guidelines document, composed of principles that its members agree to follow and recommendations made by IRDiRC Scientific Committees that offer advice on best practices for RD research.^1^ It emphasises the need for collaboration in RD research, the involvement of patients and their representatives in all relevant aspects of research, and the importance of sharing data and resources. On the basis of these principles and to contribute to its mission, IRDiRC introduced a quality indicator in March 2015 called ‘IRDiRC Recommended' to highlight for researchers' resources which, if more broadly used, are expected to accelerate advances in RD research and development.^2^

Earlier this year, this indicator was renamed ‘IRDiRC Recognized Resources'^3^ to convey more clearly the goal of the initiative: designating resources for RD research that have received recognition by researchers in the RD community. To obtain this label, resources undergo a peer-review process by IRDiRC Scientific Committee members and IRDiRC-independent researchers, who are often users of these resources themselves. The ‘IRDiRC Recognized Resources' indicator is expected to promote the pace of discoveries and translation into clinical applications, in addition to encouraging the adoption of recognised standards, guidelines, tools, and platforms that facilitate research advances guided by the principles of interoperability and sharing. This paper presents an overview of the resources that have obtained the ‘IRDiRC Recognized Resources' designation after the first year of its implementation.

## IRDiRC Recognized Resources

The application for ‘IRDiRC Recognized Resources' is open to all project leaders of different resources (ie, standards, guidelines, tools, and platforms) designed to advance RD research and development. Applications are peer-reviewed with rolling submission based on a number of criteria (Table 1), and in particular, two mandatory requirements: resources must be within IRDiRC's focus and mission as defined in the IRDiRC Policies and Guidelines document, and have multinational connectivity and audience. Eligible resources include software, bioinformatics platforms, web services, RD data or biospecimen collections, international standards, and international guidelines (Table 2). Other resources, although acknowledged as important, are not eligible for the recognition: national, regional, or institutional biobanks and registries for RD; resources that are dedicated to a single disease entity; resources that could provide some utility for RD, but are primarily designed for broader use; and commercial resources.

In its first year, 13 resources have been given the ‘IRDiRC Recognized Resources' designation. These resources include three guidelines, five platforms, two reference databases, two standards, and an advisory committee (Table 3). These resources are hereby presented according to the area of focus in the context of the goals and objectives of IRDiRC.

### Facilitating international data sharing for discovery

Four recognised resources have a strong emphasis on sharing approaches, aiming to advance knowledge about RD by encouraging global scientific collaboration while respecting ethical considerations. Two of these resources set out guidelines for legally- and ethically-grounded sharing. The ‘International Charter of principles for sharing bio-specimens and data'^4^ and the ‘Framework for responsible sharing of genomic and health-related data'^5^ aim to remove bottlenecks for effective sharing of RD data without compromising privacy, consent, and interest of individuals who participate and contribute their data for biomedical research. PhenomeCentral is a repository of RD patient phenotypic and genetic data, deposited by clinicians and scientists in this secure database to facilitate discovery; its collaborative model aims to understand the clinical spectrum and underlying mechanism of RD.^6^ In a similar manner, DECIPHER is a database that enables the depositing, analysing, and sharing of phenotype-linked plausible variation in patients with RD, thereby empowering diagnosis and discovery.^7^ Both PhenomeCentral and DECIPHER are participants of the Matchmaker Exchange initiative, a collaboration between IRDiRC and the Global Alliance for Genomics and Health^8^ to enable genomic discovery through the exchange of phenotypic and genotypic profiles using a federated platform,^9^ thereby supporting the IRDiRC goal of providing a means to diagnose most RDs by 2020.

### Knowledge organisation and ontologies

Five widely used recognised resources are found in the area of knowledge organisation and ontologies. With respect to knowledge organisation, two databases facilitate access to RD information, Orphanet, and the Online Mendelian Inheritance in Man (OMIM). Orphanet is a reference portal for RD and orphan drugs.^10^ It provides comprehensive information on research projects and their funders, registries and biobanks, platforms for research, clinical trials, a nomenclature and classifications of RDs, genes, and associated phenotypic and epidemiological data. OMIM is a widely used knowledge base of human genes and associated phenotypes comprised of over 23 000 structured free-text entries.^11, 12^ It captures published disease-gene relationships, and is a primary resource in human genetics for cataloguing and describing rare Mendelian diseases, and an essential tool for RD diagnosis.

Ontologies define a standard, controlled vocabulary for different fields in science and medicine to be utilised in data integration, organisation, search, and analysis. The Orphanet Rare Disease Ontology (ORDO), developed by Orphanet and the European Bioinformatics Institute, integrates different resources to provide a common framework for computational analysis of RD; it presents a structured vocabulary for RD, capturing relationships between diseases, genes, and other relevant features.^13^ The Human Phenotype Ontology (HPO) provides a standardised vocabulary to describe phenotypic abnormalities encountered in human disease, thus facilitating the exchange of clinical data and assisting RD diagnosis or searches for novel genes.^14^ The International Consortium of Human Phenotype Terminologies (ICHPT) has produced standard terms to enable interoperability between databases capturing clinical information,^15^ enabling connections between scientific resources for use in RD research.

### Networking patient registries

Another necessary component to enable IRDiRC's mission is patient registries, which are organised databases of patient information, a critical tool for RD research. International collaboration regarding such databases is essential to assemble sufficient numbers of patients, for example to ascertain pathogenicity of rare genotypes, to carry out natural history studies, and identify participants for research and clinical trials. TREAT-NMD Patient Registries, a global network of national registries providing a single entry point for access to rare neuromuscular disease patients worldwide, is such a recognised platform,^16, 17^ and is expected to facilitate clinical trialling for this group of RDs.

### Therapeutic development

IRDiRC aims to stimulate the development of 200 new therapies by 2020. TREAT-NMD has been active in confronting barriers in this space, producing resources to contribute to different phases of therapeutic development, from translation of basic research to clinical trials. Three additional resources from TREAT-NMD met IRDiRC's requirement regarding the adequate and timely exchange of scientific and regulatory information about clinical research, and are therefore recognised as important tools towards development of new therapies. First, its ‘Standard Operating Procedures for preclinical efficacy studies' consist of a collection of experimental protocols for the most common outcome measures used in the assessment of drug efficacy in mammalian models of rare neuromuscular diseases.^18, 19^ A wide use of such standardised protocols would contribute to improving robustness of preclinical results that serve to justify patient trials. One of the hurdles prior to initiating a clinical trial is the identification of trial sites capable of recruitment of sufficient number of patients, and qualified personnel to provide care and experience given a specific standard. The aim of the TREAT-NMD Care and Trial Site Registry is to help industry and clinical investigators identify and select trial sites and potential partners for clinical studies in neuromuscular and neurodegenerative diseases.^20^ The TREAT-NMD Advisory Committee for Therapeutics is composed of drug development experts from academia and industry, as well as representatives from patient and scientific research centres. This committee meets twice yearly to review and provide guidance on the translation and therapeutic development path for rare neuromuscular diseases with large unmet needs.^21^ Wide-spread adoption by the community of such recognised resources is expected to facilitate the IRDiRC goal to enable the development of 200 new therapies by 2020.

## Conclusion

After its first year, an assessment of the ‘IRDiRC Recognized Resources' indicator shows that this initiative has highlighted 13 resources that focus on key IRDiRC priorities in advancing RD research: data sharing for discovery, knowledge organisation and ontologies, networking patient registries, and therapeutic development. It is expected that as more resources are highlighted and used by researchers, the pace of discovery and translation into the clinic will be further enhanced. Reciprocal recognition of resources between international efforts with overlapping goals (e.g. Human Variome Project) will be an important step forward. Moreover, as the research community converges on and adopts recognized standards, guidelines, tools, and platforms, the resulting interoperability will enable enhanced sharing, and thus accelerate advances in research and development across all RD.

## Figures and Tables

**Table 1:** Assessment criteria for ‘IRDiRC Recognized Resources'

*Mandatory criteria*	*Additional criteria*
Within IRDiRC's focus and mission	Functional and accessible with minimal downtime
Multinational connectivity and audience	Development and maintenance team
	Clear and well-documented terms of use and license policies
	Adheres to all relevant ethical and privacy policies and requirements
	Process in place for quality control and life cycle management
	Undergoes scientific peer review
	Financially viable for the following 3 years
	Documents its core impacts (eg, number of users, number of visits, etc)
	Demonstrates relevant and ongoing activity in sharing and dissemination

**Table 2:** Eligibility for different types of ‘IRDiRC Recognized Resources'

*Eligible resources*	*Excluded resources*
Software, bioinformatics platforms, and web services	National, regional, or institutional biobanks RD, or a single disease entity
Data collections/biospecimen collections	National, regional, or institutional registries for RD, or a single disease entity
International standards	Resources with some utility for RD research, but primarily designed for broader use
International guidelines	Commercial resources

**Table 3:** IRDiRC Recognized Resources

*Resource name*	*Type*	*Description*
*Facilitating international data sharing*		
International Charter of principles for sharing bio-specimens and data	Guideline	The Charter provides recommendations for successful legally- and ethically-grounded sharing of bio-specimens and data
Framework for responsible sharing of genomic and health-related data	Guideline	The Framework provides a principled and practical framework for the responsible sharing of genomic and health-related data
PhenomeCentral	Platform	PhenomeCentral is a repository for secure sharing of phenotypic and genotypic data in the RD community, thereby connecting patient profiles
DECIPHER	Platform	DECIPHER is a database and web-based platform enabling the deposition, analysis, and sharing of phenotype-linked plausibly pathogenic variation in patients with RD
		
*Knowledge organisation and ontologies*		
Orphanet	Reference/database	Orphanet is a reference portal for information on RD and orphan drugs
Online Mendelian inheritance in man (OMIM)	Reference/database	OMIM is a database of human genes and genetic phenotypes comprised of over 23 000 structured free-text entries
Orphanet rare disease ontology (ORDO)	Platform	ORDO provides a structured vocabulary for RD, thereby aiming to define relationships between diseases, genes, and other features of interest
Human phenotype ontology (HPO)	Standard	HPO provides a standardised vocabulary of phenotypic abnormalities encountered in human disease
International Consortium of Human Phenotype Terminologies (ICHPT)	Standard	The ICHPT provides the community with a set of terms to describe phenotypic features to be used by any terminologies to achieve interoperability between databases, in particular, to allow the linking of phenotype and genotype databases for RDs
		
*Networking patient registries*		
TREAT-NMD patient registries	Platform	The TREAT-NMD Patient Registries is a global network of national registries that provides a unique entry point for access to rare neuromuscular disease patients worldwide
		
*Therapeutic development*		
Standard operating procedures for preclinical efficacy studies	Guideline	Standard operating procedures for preclinical efficacy studies are a compilation of experimental protocols to measure drug efficacy in models of neuromuscular disease
Care and Trial Site Registry	Platform	The Care and Trial Site Registry aims to assist pharmaceutical industry and clinical investigators in deciding on clinical trial site location and in the identification of potential partners for future research projects
TREAT-NMD Advisory Committee for Therapeutics	Advisory Committee	TREAT-NMD Advisory is a group of experts from various origins (academic, industry drug development, patient representatives, and governmental representatives) that provide guidance on the translation of therapeutic programs in rare neuromuscular diseases
